# The Effects of Soil Acidity and Aluminium on the Root Systems and Shoot Growth of *Lotus pedunculatus* and *Lupinus polyphyllus*

**DOI:** 10.3390/plants13162268

**Published:** 2024-08-15

**Authors:** Lucy E. Bell, Jim L. Moir, Alistair D. Black

**Affiliations:** Faculty of Agriculture and Life Sciences, Lincoln University, Lincoln 7647, New Zealand; jim.moir@lincoln.ac.nz (J.L.M.);

**Keywords:** aluminium toxicity, forage legume, pH, root morphology

## Abstract

*Lotus pedunculatus* (lotus) and *Lupinus polyphyllus* (Russell lupin) persist in the upland grasslands of New Zealand, where soil acidity and associated aluminium (Al) toxicity impede conventional pasture legumes. This experiment investigated the response of lotus and Russell lupin to soil acidity and Al. The species were sown in 20 cm tall 1.2 L pots of acidic upland soil. A mass of 4.5 or 6.7 g lime (CaCO_3_)/L was added to either the top or bottom or both soil horizons (0–9 cm and 9–18 cm), resulting in six treatments across six randomised blocks in a glasshouse. The soil pH was 4.4, 4.9, and 5.4; the exchangeable Al concentrations were 24, 2.5, and 1.5 mg/kg for 0, 4.5, and 6.7 g lime/L. At 16 weeks post-sowing, the plants were divided into shoots and roots at 0–9 cm and 9–18 cm. Root morphology, shoot and root dry matter (DM), shoot nitrogen (N), and nodulation were measured. The total plant DM and shoot-to-root DM ratio were higher, and the shoot %N was lower for the lotus plants than the Russell lupin plants for the various lime rates (13.2 vs. 2.9 g plant^−1^, 5.6 vs. 1.6, and 2.4 vs. 3.3%, *p* < 0.05). No response to lime in terms of total DM or total root morphology parameters was exhibited in either species (*p* > 0.05). Root morphology adjustments in response to acidity between soil horizons were not observed. The results indicated that lotus and Russell lupin are tolerant to high soil acidity (pH 4.4–5.4) and exchangeable Al (1.5–24 mg kg^−1^), highlighting their considerable adaptation to grasslands with acidic soils.

## 1. Introduction

Globally, forage and arable crop scope and productivity are strongly limited by soil acidity. An estimated 30% of the world’s ice-free land is acidic, with soils having a pH of 5.5 or less [1]. A low soil pH limits plant productivity by several mechanisms, including reduced nutrient availability and increased solubility and bioavailability of trace elements [2]. A fundamental limitation when soil pH decreases below 5.5 is the mobilisation of exchangeable aluminium (Al), which can cause Al toxicity in plants [3]. At low pH, soil Al exists in various forms. However, Al^3+^ is considered to be the major phytotoxic form [4]. Toxic concentrations of Al adversely affect plant persistence and production by inducing a series of morphological and physiological changes [5]. One of the earliest indicators of Al stress is inhibited root growth [6]. High concentrations of Al primarily affect the root apex by reducing cell elongation and division, as well as root hair development. As a result, root growth is stunted. Furthermore, lateral root growth has been associated with plants selectively growing away from toxic concentrations of Al [7]. Damage to roots and growth stunting adversely impact the ability of plants to access nutrients and water in the soil profile, resulting in abiotic stress [6]. Importantly, root nodule formation in legumes can be reduced in acidic soils, as Al toxicity can impair rhizobia abundance and survival [8].

Soil acidification and associated Al toxicity have been identified as limitations to pastoral farming sustainability and productivity in the uplands (hill and high country) of New Zealand [9]. Legumes play a critical role in these farming systems due to their ability to fix biological nitrogen, which drives grassland production [10]. This process results in the supply of nitrogen (N) to grasses in grasslands, thus improving total dry matter (DM) production [11]. Furthermore, legumes provide high-quality feed to grazing animals [12,13].

Traditionally sown legumes such as white clover (*Trifolium repens* L.) fare poorly under the hill and high country’s typical acidic soils and climate. The amelioration of soil acidity by lime application is often not economical due to remote locations and high aerial application costs [14]. Additionally, soil pH can vary through the soil profile [15], making soil acidity management challenging for farmers due to lime’s low solubility and passive movement [16]. Surface applications are ineffective at increasing soil pH below the shallow top layer.

Legumes differ considerably in their tolerance to soil acidity and exchangeable Al [17]. However, only a very limited number of forage legumes are viable for farmers to implement in upland hill and high country environments. Two perennial legume species, lotus (*Lotus pedunculatus* Cav.) and Russell lupin (*Lupinus polyphyllus* Lindl.), have been identified as legume species of interest for acidic soils [16,18]. Lotus is a rhizomatous species which develops an extensive root system consisting of a taproot and a network of fibrous roots [19,20]. Its root growth accumulation occurs during late summer and autumn, resulting in a large increase in root dry matter. Its shoot growth is characterized by broadly obovate and obtuse leaflets on vegetative shoots [19]. Russell lupin’s growth characteristics include the development of a taproot and growth from a central crown [21,22]. Vegetative digitate leaves are extended from the crown by large petioles [22].

As the primary symptom of Al toxicity is the inhibition of root growth, it is important to understand the root morphology of these species at varying soil pH and exchangeable Al concentrations. It is of interest to consider how a variation in soil pH with depth causes adjustments or compensations in root morphology. The uniformity of soil chemistry with increasing depth does not always occur under field conditions. Therefore, investigating root morphology variations due to changing soil pH and Al concentrations with soil depth is warranted, as it may provide insight into species’ tolerance mechanisms for acidity. The glasshouse experiment described in this manuscript aimed to assess the influence of soil pH and Al on the root biomass, morphology, and nodulation of lotus and Russell lupin.

## 2. Results

### 2.1. Soil pH, Exchangeable Al, and Oxalate-Extractable Al

**Figure 1:**
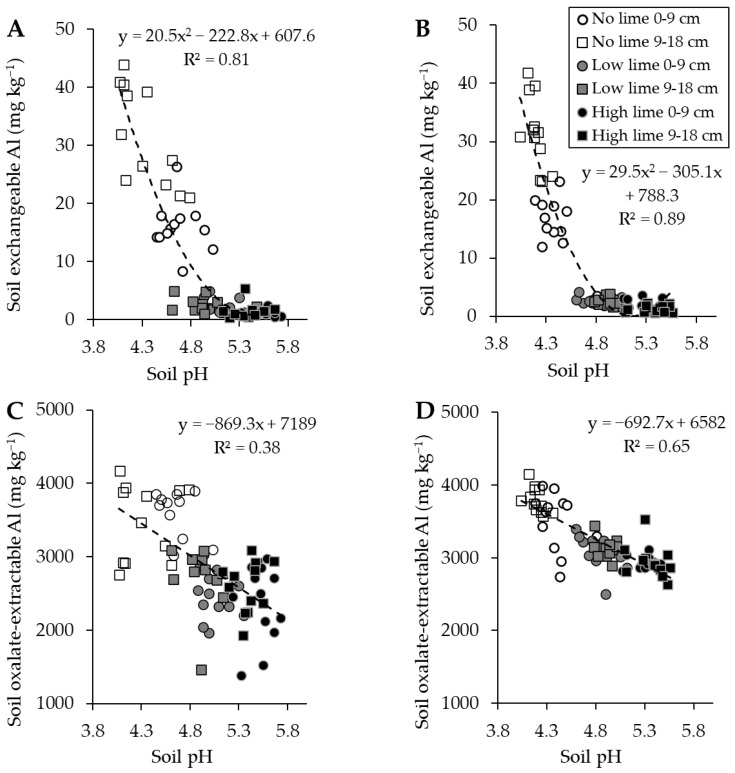
Relationship between soil pH and soil exchangeable Al (mg kg^−1^) for (**A**) lotus (*p* < 0.001) and (**B**) Russell lupin (*p* < 0.001), and the relationship between soil pH and oxalate-extractable Al (mg kg^−1^) for (**C**) lotus (*p* < 0.001) and (**D**) Russell lupin (*p* < 0.001).

The lime additions increased the soil pH from a mean of 4.4 (when no lime was added) to 4.9 (when a low lime rate was added) and 5.4 (when a high lime rate was added). The relationships between soil pH and soil exchangeable Al are best described as polynomial (Figure 1A, lotus R^2^ = 0.81; B, Russell lupin R^2^ = 0.89). Significant decreases in soil exchangeable Al to 2.5 and 1.5 mg kg^−1^ were observed for the additions of the low and high lime rates, respectively (*p* < 0.05). A key difference observed for the treatment without added lime was lower mean concentrations of exchangeable Al in the 0–9 cm horizon (16 mg kg^−1^) compared with those for the 9–18 cm horizon (32 mg kg^−1^) (Figure 1A,B) and those for the control without added lime (27 mg kg^−1^) (*p* < 0.05).

In contrast to the exchangeable Al, the relationships between soil pH and oxalate-extractable Al are best described as poor to moderate (Figure 1C, lotus R^2^ = 0.38; D, Russell lupin R^2^ = 0.65).

### 2.2. Shoot Biomass and Shoot Nitrogen

The mean shoot biomass for the lotus plants with differing lime rates was 11.1 g DM plant^−1^, which is 6.17-fold greater than that of the Russell lupin plants, which was 1.8 g plant^−1^ (Table 1; *p* < 0.05). The mean shoot %N of the Russell lupin plants was greater than the lotus plants by 0.9% (Table 1; *p* < 0.05). For each legume species, neither the shoot biomass nor shoot N responded to the lime additions (Table 1; *p* > 0.05).

### 2.3. Root Morphology

The investigation of total plant root parameters (the sum of the 0–9 cm and 9–18 cm horizons) indicated large differences between the two legume species (Table 1). The mean total root biomass of the lotus plants (2.0 g DM plant^−1^) was greater than that of the Russell lupin plants (1.1 g DM plant^−1^) (*p* < 0.05). Furthermore, the lotus plants’ root length and surface area were 3.86- and 3.01-fold greater than those of the Russell lupin plants (*p* < 0.05). However, the Russell lupin plants had a greater average root diameter (*p* < 0.05). Within each legume species, there were no significant differences in the total root parameters between lime treatments (*p* > 0.05).

In contrast to total root parameters, an analysis of root biomass, length, surface area, and average diameter by horizon depth indicated differences between the 0–9 cm and 9–18 cm horizons and with the different lime rates (*p* < 0.05, Table 2). The species by treatment interaction for all root parameters (*p* < 0.05) bar the average diameter (*p* > 0.05) demonstrates that the legume species’ root parameters at each horizon depth responded differently to increasing lime rates (Table 2).

Generally, the root DM biomass was greater for both legume species in the 0–9 cm horizon than in the 9–18 cm horizon (Table 2). The lotus root biomass did not respond to the lime at each horizon depth (*p* > 0.05). A greater root biomass was shown when no lime or a low lime rate was applied in the 0–9 cm horizon of the Russell lupin plants (*p* < 0.05). However, in the 9–18 cm horizon, the greatest root biomass was under the treatments without lime or with a high lime rate (*p* < 0.05). Consideration is required that the high lime rate treatment in the 9-18 cm horizon was associated with no lime in the 0–9 cm horizon.

The lotus root length and surface area did not differ between depth horizons or with the rates of lime addition (*p* > 0.05, Table 2). The Russell lupin plants’ root morphology exhibited differences (*p* < 0.05, Table 2). Generally, the Russell lupin 0–9 cm horizon had a greater root length and surface area than the corresponding 9–18 cm horizon. Notably, the largest root length and surface area were present at 0–9 cm for the treatments with no or low lime rates, consistent with the results of the root biomass (*p* < 0.05). The root length and surface area in the 9–18 cm horizon were greater in treatment 6B (in which a high lime rate was added) at 696 cm and 125 cm^3^, respectively, than in treatments 4B (in which no lime was added) and 2B (in which a high lime rate was added) (*p* < 0.05). Notably, 4B and 2B correspond to high lime contents in the 0–9 cm horizon.

Unlike other root parameters, no interaction between species and treatment existed for the average root diameter (*p* = 0.24, Table 2). Across both legume species, the average diameter was the greatest in the Russell lupin plants at 0–9 cm (Table 2). Unlike the root length and surface area, the Russell lupin plants’ average diameter did not respond to the lime at 0–9 cm (*p* > 0.05). Generally, this was also observed at 9–18 cm. Similarly to the root DM biomass, there was no change in the average diameter for the different additions of lime within each horizon for the lotus plants (*p* > 0.05).

### 2.4. Nodulation

**Figure 2:**
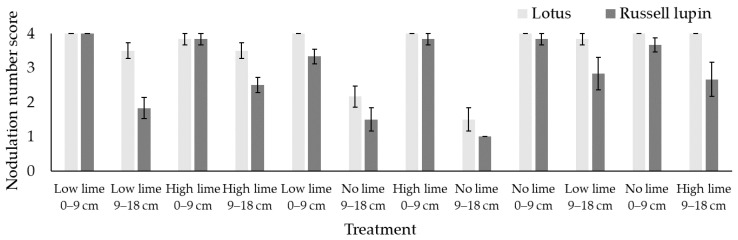
Nodulation number score of lotus and Russell lupin. Bars represent mean ± SEM.

The lotus plants had a mean nodulation score of 3.4 out of 4, which was greater than that of the Russell lupin plants, which had a score of 2.6 (*p* < 0.001). The lotus nodulation did not change across the lime treatments in the 0–9 cm horizon, with a mean nodulation score of 4 (Figure 2). However, in the 9–18 cm horizon, the lotus nodulation score was lower in pots in which no lime was applied (1.9) compared to those in which lime was applied (3.7). The Russell lupin plants generally had similar nodulation to the lotus plants in the 0–9 cm horizon (3.1) (Figure 2); however, the 9–18 cm horizon had consistently lower nodulation scores (2.1).

### 2.5. Relationships

**Figure 3:**
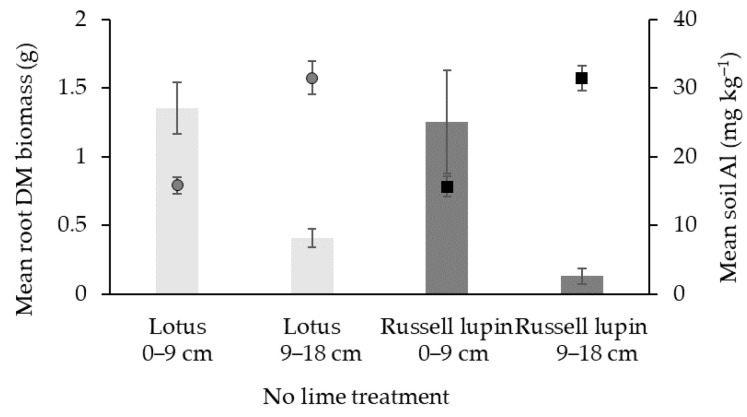
Mean root DM biomass (g plant^−1^) (bars) of lotus and Russell lupin under the treatment in which no lime was added and soil exchangeable Al (mg kg^−1^) (points). Bars and points represent mean ± SEM.

In the treatment in which no lime was added, a higher root DM biomass and lower soil exchangeable Al concentrations were observed in the 0–9 cm horizon; in comparison, the inverse is shown in the 9–18 cm horizon of both species (Figure 3). The mean soil exchangeable Al was 16 mg kg^−1^ in the 0–9 cm horizon for both species. However, in the 9–18 cm horizon, the mean soil exchangeable Al concentrations were 32 mg kg^−1^ and 31 mg kg^−1^ for the lotus and Russell lupin plants, respectively.

**Figure 4:**
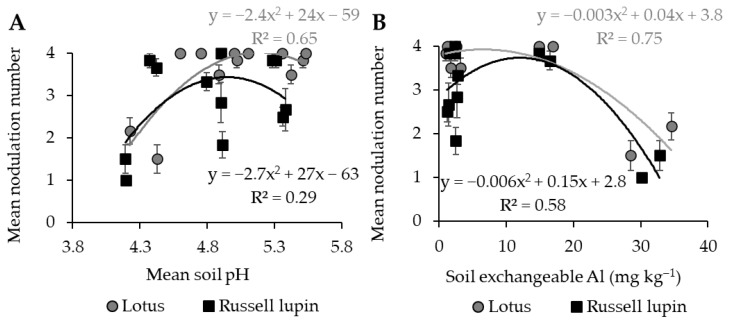
Relationships between mean nodulation number score and (**A**) soil pH and (**B**) soil exchangeable Al of lotus and Russell lupin plants. The grey and black lines represent the relationships of lotus and Russell lupin, respectively. Points represent mean nodulation number ± SEM.

A polynomial relationship was observed between the mean soil pH and mean nodulation number score for the lotus plants (Figure 4A, R^2^ = 0.65). The nodulation score was halved as the soil pH decreased from 4.6 to 4.3. In contrast, a poor polynomial relationship was observed between the mean soil pH and Russell lupin nodulation score (Figure 4A, R^2^ = 0.29). Polynomial relationships were observed between the mean soil exchangeable Al and nodulation score for the lotus (R = 0.75) and Russell lupin (R = 0.58) plants (Figure 4B). A sharp decline in nodulation with an increase in soil exchangeable Al from 15 to 30 mg kg^−1^ was shown in both species.

## 3. Discussion

This study found that the lotus and Russell lupin plants were very tolerant of extreme acidic soil conditions within a soil pH range of 4.4–5.4. Their lack of response to the addition of lime in terms of the parameters of shoot biomass, total root biomass, and total root morphology illustrated these legume species’ tolerance to low soil pH. Moreover, detrimental effects, adjustments or compensations in root morphology in response to greater soil acidity in the 0–9 or 9–18 cm horizons were not observed.

Acidic soils can limit the productivity of sensitive pasture and crop legumes due to the mobilisation of toxic metals such as Al, reduction in plant root access to essential nutrients and water [23], and inhibition of the survival of rhizobia [8]. Sensitive legume species such as white clover (*Trifolium repens* L.) have demonstrated intolerances to Al when concentrations exceed 3 mg kg^−1^ [17,24]. Such phytotoxic concentrations of Al are generally associated with soil pH below 5.5 [3]. Despite subjecting the lotus and Russell lupin plants to very acidic soils (pH < 5.5) and concentrations of exchangeable Al known to be toxic to other legume species, no adverse effects were observed in terms of shoot and root biomass. This observed plant tolerance within the 4.4–5.4 pH range underscores the adaptability of these species to acidic soil environments.

A strong relationship between soil pH and exchangeable Al was established for this soil. Morton and Moir [9] reported that a range of New Zealand soils had consistently increased soil exchangeable Al concentrations as the soil pH decreased. The addition of lime to the very acidic soil used in this experiment resulted in significant decreases in soil Al despite an acidic soil pH of less than 5.5, thus indicating a substantial effect of lime on soil Al bioavailability.

The lime additions reduced oxalate-extractable Al concentrations, which were associated with soil pH. In our other experiments at Lincoln University (in progress), soil oxalate-extractable Al and organically complexed Al have been revealed as key controls of Al solubility. In this experiment, the relationship between soil pH and Al fractions was stronger with the exchangeable Al than with the oxalate-extractable Al.

The relationship between soil pH and exchangeable Al and oxalate-extractable Al was weaker for the soil of the lotus plants than that of the Russell lupin plants, which indicates the plant species has a possible effect on Al concentrations. A key finding was the approximately 50% reduction in exchangeable Al in the 0–9 cm horizon compared with the 9–18 cm horizon and the control soil of the pots without lime for both legume species. The lower exchangeable Al concentrations are correlated with higher soil pH in the 0–9 cm horizon. Furthermore, the large reduction in Al may be a mechanism for Al tolerance in plants. It is possible that given an extended experimental period, an increase in pH and reduction in soil Al may have been observed in the 9–18 cm horizon.

It has been reported that some plants can influence rhizosphere soil by excreting root exudates [25,26]. These exudates can include organic acids such as malate, citrate, and oxalate, which chelate Al in the soil solution of acid soils, thus rendering Al non-toxic [27]. Malate and citrate have been identified to be excreted in large quantities relative to other organic acids under Al stress by some Al-tolerant species [28]. For example, high malate concentrations have been found in the annual legume Serradella species and attributed to its tolerance to soil Al [29,30]. In addition, malate and citrate were found in the highest quantities of all organic acids analysed in the rhizosphere of Russell lupin when grown in acidic soil [31]. In contrast to the findings of this study, Bouray et al. [31] did not report a reduction in soil exchangeable Al in pots of Russell lupin without lime when grown in acidic New Zealand soil, compared to the initial soil test results. However, their initial soil exchangeable Al concentrations were much lower (5.8 mg kg^−1^) than in this study.

Stoutjesdik et al. [32] found lotus rendered Al non-toxic at the root tips by forming Al–tannin complexes. However, the authors noted that further research is required before the release of tannins can be established as an Al tolerance mechanism of lotus plants or other plant species. To our knowledge, no studies have investigated organic acids as a potential Al tolerance mechanism of lotus. Analyses of the exudates of lotus are required to explore the possible reason for the reduction in soil exchangeable Al.

In addition to the possible effect of plants on the concentrations of exchangeable Al through exudates, Al–organic complexes may explain some of the difference in exchangeable Al between the 0–9 cm and 9–18 cm horizons. Generally, the root mass was greater in the 0–9 cm horizon than in the 9–18 cm horizon. The greater root mass in the 0–9 cm horizon may have increased the soil organic matter [33], and in association with higher soil pH, enabled the greater complexation of soluble Al to Al–organic complexes [34].

An important finding of this study was that the shoot and total root biomass did not respond to lime additions across the pH range of 4.4 to 5.4 for either legume species. Lotus has previously been identified as a legume species of interest in acidic soils, having had better yields than other perennial legume species in acidic high country soils in glasshouse experiments [17,35]. These studies found lotus shoot DM biomasses of 8.2 g DM pot^−1^ (at a pH of 5.0) and 8.3 g DM pot^−1^ (at a pH of 5.4) over 10- and 11-month growth periods without lime, with basal nutrient application, and with plant densities of five plants per pot. In contrast, a higher DM biomass was observed in the current study over a shorter experimental period of four months. This difference may be attributed to plant numbers per pot or pot size restricting the root biomass and therefore the shoot biomass in previous studies [36].

Lower shoot DM biomasses ranging from 1900 to 3200 kg DM ha^−1^ year^−1^ have been observed in field trials at upland sites (at pH of 4.7 and 4.9) [18]. This indicates that climate and environmental factors may be the key restrictors of lotus shoot biomass at upland locations rather than soil acidity, as demonstrated by the lotus plants’ lack of response to lime at a low soil pH in this study.

In contrast to our findings, a previous glasshouse experiment found that lotus root biomass responded to lime applications [37]. This may be explained by the lower initial soil pH of 4.2 used in that study. The greater root biomass in the 0–9 cm horizon compared with the 9–18 cm horizon of both legume species is likely related to root physiology rather than being a treatment effect, as demonstrated by the lack of response to lime within the horizons. Differences between the two horizons reflect plants’ development in the shallower horizon before expanding into the deeper layer. A further consideration of this study is that moisture was not limited, and water was applied at the top of the pot, which may have resulted in a lower root biomass, length, and surface area in the lower soil horizon, as the plants did not need to grow deeper to access soil moisture. Plant roots must explore the soil profile deeper to access soil moisture in a natural field setting, which may present different results.

Variations in soil pH and soil Al down the soil profile may cause changes to root depth and morphology. However, no differences in root length or surface area were observed with differing soil pH and Al concentrations across the two depths analysed for the lotus plants. To our knowledge, these lotus root morphology parameters have not been investigated in acidic soils. These new findings regarding the absence of a correlation between lotus root morphology and soil pH provide a quantification of lotus’s strong tolerance to extreme soil pH and high exchangeable Al concentrations.

The comparable shoot DM biomass of the Russell lupin plants and the lack of response to the lime applications are consistent with the findings of a glasshouse experiment [31] and a field trial [16]. However, compared with the previous literature, the very acidic pH range observed in the current study presents new information regarding Russell lupin’s tolerance to soils with a soil pH less than 5.0. The six-fold greater shoot biomass of lotus compared to Russell lupin may partly be explained by their differing plant physiology and growth habits. Russell lupin grows from a single crown, with stems forming one single leaflet [22], in contrast to lotus, which develops many vegetative shoots and spreads by stolons and shallow rhizomes [20], which were observed in this study.

Unlike the lotus plants, differences in root biomass were found between lime rates at each depth horizon of the Russell lupin plants. The largest root biomass was observed in the plants without lime at the 0–9 cm depth and with the addition of a high lime rate at the 9–18 cm depth. However, the greater biomass likely reflected the early germination time of two of the six replicates. In contrast, differences in root length and surface area within the 0–9 cm horizon were not correlated with germination timing. The treatments without lime or with low lime addition rates showed greater root lengths and surface areas. This further demonstrated Russell lupin’s tolerance to acidic soil conditions. Hendrie et al. [16] found Russell lupin root biomass and nodulation did not change in response to lime within the pH range of 5 to 6.8 and an exchangeable Al concentration less than 15 mg kg^−1^. In addition, wild Russell lupin has been found to be growing across strongly to weakly acidic sites in Sweden and central Europe [22]. The lack of response to lime indicates that Russell lupin has adaptive traits to very acidic soils with high Al concentrations.

Some rhizobia vary in their sensitivity to acidic soils [38]. However, it has previously been demonstrated that acidic conditions were not detrimental to nodulation in lotus [39,40]. A strong relationship between soil exchangeable Al and nodulation for lotus was observed. Nodulation was reduced at the 9–18 cm depth when no lime was applied. In contrast, at the 0–9 cm depth, there was no difference in nodulation between treatments. This is likely a result of the 50% reduction in soil exchangeable Al in the 0–9 cm horizon compared with the 9–18 cm horizon in the treatment without lime.

The nodulation of the Russell lupin plants was greater in the 0–9 cm horizon compared to the 9–18 cm horizon. However, their nodulation within horizons did not differ with the lime treatments. Thus, the tolerance of rhizobia to acidic conditions and high Al concentrations is further quantified, as shown in previous research [16]. The shoot N did not differ between the lime treatments of either legume species. Therefore, the observed differences in nodulation did not influence the shoot N.

## 4. Materials and Methods

### 4.1. Soil Collection and Soil Chemical Analysis

Soil (0–0.15 m depth) was collected from Avenel Station (S45°36.78′E169°35.62′; 861 m.a.s.l; 900 mm annual rainfall), which is located near Millers Flat, Central Otago, New Zealand, in June 2021. The soil is classified as a brown soil (New Zealand Soil Classification [41]; the United States Department of Agriculture classification: Dystrudepts [42]). The collected soil was air-dried (20° C) and sieved (4 mm for experiment and 2 mm for analyses). Soil fertility status was determined and is presented in Table 3.

### 4.2. Experimental Design and Treatments

**Figure 5:**
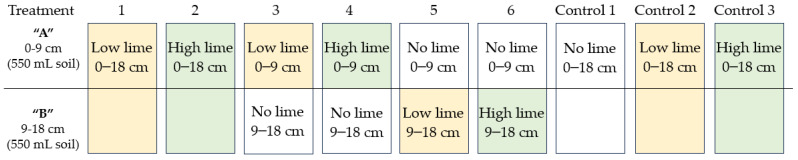
Illustration of full pot and split pot lime treatments and controls.

A pot experiment was conducted in a temperature-controlled glasshouse at Lincoln University (Lincoln, New Zealand) over a 16-week summer–autumn period from January to May 2023 under natural light conditions. Average daytime and night-time temperatures were 20 °C/18 °C, respectively. The experiment was a completely randomised block design consisting of six treatments and six replicates. The six treatments comprised two full pots and four split pot treatments, as shown in Figure 5. Additionally, three full pot control treatments were included: without lime, with a low lime rate, and with a high lime rate, each with six replicates, and no plants.

Based on previous research on the Avenel soil, high and low lime rates of 6.7 mg CaCO_3_ mL^−1^ to increase pH to 5.8 and 4.5 mg CaCO_3_ mL^−1^ to increase pH to 5.1, respectively, were applied [49,50]. Basal nutrients were applied to each pot at the rates of 0.1 g P L^−1^ (Ca (H_2_PO_4_)^2^·H_2_O Monohydrate, 24.6% P), 0.9 g S L^−1^ (CaSO_4_·2H_2_O, 18.6% S), and 0.4 g K L^−1^ (KCl, 50% K). The application of 0.1 g P L^−1^ likely resulted in an increase in Olsen P to greater than 20, as shown for a similar soil [17]. This Olsen P is correlated with approximately 90% relative yield for lotus and Russell lupin [17,51,52]. Lime and nutrients were thoroughly mixed with 1100 mL of soil for the full pot and 550 mL for the half-pot treatments before being placed in 1200 mL pots.

### 4.3. Trial Management

Prior to sowing, Russell lupin seed was scarified in 97% sulphuric acid (H_2_SO_4_, Scharlau, 1.84 g cm^−3^) at 2:1 ratio of acid to seed for 20 min, followed by 20 min of rinsing with deionised water [53]. Based on germination testing, five Russell lupin seeds per pot and two lotus (cv. Trojan) seeds per pot were sown on 22 January 2023. Additional Russell lupin seeds were sown until there was one plant per pot. Pots were thinned to one plant per pot as required. On 28 January 2023, pots were inoculated with fresh, ground functional nodules collected from field-grown lotus and Russell lupin. Pot soil moisture content was maintained at 32% Vw/Vt by an automated irrigation system with six calibrated soil moisture sensors (Decagon 5TM, Decagon Devices LTD, USA). The irrigation control system consisted of a Campbell Scientific CR23X data logger.

### 4.4. Measurements and Analysis

Plants were harvested 16 weeks after sowing in May 2023 and removed from pots. Root systems were cut in half at a depth of 9 cm (to reflect the soil lime treatments). Soil and root samples were separated into horizons A (0–9 cm) and B (9–18 cm). Shoot material was removed from the crown. The soil was removed from each half-root system, labelled ‘A’ (0–9 cm) and ‘B’ (9–18 cm), and air-dried for seven days before further analysis. Root systems were washed in water, and root nodulation was scored according to a modified method by Rice et al. [54]. Nodule characteristics of number, position, and size were scored between 1 and 4, and colour was scored between 1 and 6. Within each legume species, nodule position, size, and colour were relatively uniform across treatments. Consequently, the number of nodules was determined to be the key factor responsible for the fluctuations in the overall nodule score. As a result, the analysis focused exclusively on the nodulation number score as a categorical variable. The nodulation number was scored from 1 (no nodules) to 4 (20+ nodules).

Root systems were then carefully stored flat in plastic bags with deionised water and stored at 4 °C until root scanning. Root samples were scanned using an Epson flatbed scanner (Epson Perfection V850 Pro), and the scanned images were analysed using WinRhizo (Pro version, 2022). When there was a large root density, roots were scanned over two trays, and values were summed for each tray or averaged for the root diameter. Following scanning, the roots were oven-dried at 70 °C for 48 h and weighed. Herbage samples were oven-dried at 70 °C for 48 h, weighed, and finely ground prior to analysis of total N by combustion using a Vario-Max CN Elemental analyser (Elementar GmbH, Hanau, Germany). The shoot biomass data in this paper are presented as g per m^2^ scaled from the surface area of the pots, expressed using the standard commonly used unit of g per m^2^. All dried soil samples were sieved (2 mm) and analysed for pH (1:2.5, soil:deionised water ratio [43]) using a pH probe (SevenEasy pH meter, Mettler Toledo, USA). Exchangeable Al was extracted using 0.02 M CaCl_2_ (1:2 soil:extractant ratio) [48,55]; oxalate-extractable Al, Fe, and Si were extracted using an acid ammonium oxalate reagent (0.1:10 soil:extractant ratio) [43,56]; and extracts were analysed using an ICP-OES (Varian 720-ES ICP-OES, Varian, Melbourne, Australia).

### 4.5. Statistical Analysis

The analysis was performed using Genstat (version 23, VSN, International). Data from each horizon (0–9 cm and 9–18 cm) were summed or averaged to analyse the total plant root parameters. The total root parameters and shoot data were analysed using a two-way analysis of variance (ANOVA) (with blocks) to assess the effects of lime treatment, species, and their interaction. One-way ANOVA was used to analyse the individual species. A linear mixed model accounting for pot/horizon depth (0–9 cm or 9–18 cm) as a random variable was applied to analyse the differences between liming treatments and species at different depths. Mean separation was performed using Fisher’s unprotected LSD (5%). When applicable, the data were transformed using logbase10. A generalised linear mixed model (Poisson distribution) was used to analyse the nodulation number score. Relationships were fitted based on best fit according to R^2^ values.

## 5. Conclusions

Lotus and Russell lupin demonstrated strong tolerance to soil acidity (4.5–5.4) and soil exchangeable Al (1.5–24 mg kg^−1^). Their lack of response in total DM biomass or root morphology parameters across the pH range studied indicated the presence of adaptive traits to acidic soils and high concentrations of exchangeable Al. Furthermore, detrimental effects or root morphology adjustments or compensations in response to different soil pH and exchangeable Al concentrations at 0–9 or 9–18 cm depths were not observed. The lotus plants exhibited a mean shoot DM biomass of 11.1 g DM plant^−1^, with a greater yield than the Russell lupin plants, which produced a mean 1.8 g DM plant^−1^. Further, the lotus plants’ mean total root DM biomass (2.0 g DM plant^−1^) and root parameters such as total root length (5687 cm plant^−1^) exceeded those of the Russell lupin plants, which yielded 1.1 g plant^−1^ and 1473 cm plant^−1^ for the mean total root biomass and length, respectively. A key result of interest was the reduction in soil exchangeable Al to 16 mg kg^−1^ in the 0–9 cm horizon, compared with the 9–18 cm horizon (32 mg kg^−1^) and the control soil (27 mg kg^−1^), in which no lime was applied, in both species. This suggests the reduction in exchangeable Al was an effect of the plants and warrants further investigation. In contrast to root morphology, reductions in nodulation number score were associated with increased exchangeable Al concentrations. However, this was not reflected in shoot N concentrations. The mean shoot %N was 2.4% and 3.3% for the lotus and Russell lupin plants, respectively, and no differences were shown across the lime treatments.

## Figures and Tables

**Table 1:** Lotus and Russell lupin grand mean for shoot dry matter (DM) biomass (g plant^−1^), shoot dry matter (DM) biomass (g m^2−1^), shoot nitrogen (N) (%), total root DM biomass (g plant^−1^), total root length (cm plant^−1^), total surface area (cm^2^ plant^−1^), average root diameter (mm plant^−1^), shoot:root ratio, and total biomass (shoot and root DM biomass, g plant^−1^) ± SEM.

	Species		Significance		
	Lotus	Russell Lupin	Species	Lime Rate	Sp*Lime
Shoot DM biomass (g DM plant^−1^)	11.1 ± 0.53	1.8 ± 0.19	*	ns	ns
Shoot DM biomass (g DM m^2−1^)	501 ± 24	73 ± 7.5	*	ns	ns
Shoot %N	2.4 ± 0.05	3.3 ± 0.13	*	ns	ns
Total root DM biomass (g plant^−1^)	2.0 ± 0.14	1.1 ± 0.23	*	ns	ns
Total root length (cm plant^−1^)	5687 ± 357	1473 ± 221	*	ns	ns
Total surface area (cm^2^ plant^−1^)	816 ± 51	265 ± 36	*	ns	ns
Average root diameter (mm plant^−1^)	0.49 ± 0.01	0.61 ± 0.02	*	ns	ns
Shoot:root ratio	5.6 ± 0.35	1.6 ± 0.19	*	ns	ns
Total biomass (g plant^−1^)	13.2 ± 0.62	2.9 ± 0.40	*	ns	ns

ns not significant at *p* < 0.05; * significant at *p* < 0.05.

**Table 2:** Mean root DM biomass (g), root length (cm), surface area (cm^2^), and average diameter (mm) of lotus and Russell lupin across different horizon depths (A 0–9 cm; B 9–18 cm) and for differing lime rates ± SEM. Means with different letters within a column differ according to an LSD multiple means separation test (*p* < 0.05).

Treatment	Root DM Biomass (g)			Root Length (cm)			Surface Area (cm^2^)			Average Diameter (mm)		
Lotus												
1A Low lime	1.87	±0.17	j	2848	±326	i	429	±48	k	0.48	±0.01	abcdefg
1B Low lime	0.53	±0.11	def	2723	±456	i	373	±69	jk	0.43	±0.01	a
2A High lime	1.71	±0.24	ij	2896	±285	i	434	±53	k	0.47	±0.02	abcdefg
2B High lime	0.60	±0.12	defg	3772	±728	i	483	±85	k	0.47	±0.06	abcde
3A Low lime	1.57	±0.19	ghij	3584	±855	i	489	±106	k	0.58	±0.08	eghijklm
3B No lime	0.53	±0.1	def	2853	±430	i	425	±68	k	0.47	±0.02	abcdef
4A High lime	1.16	±0.24	efghij	2402	±321	hi	359	±48	jk	0.47	±0.02	abcdefg
4B No lime	0.29	±0.06	d	2040	±381	hi	292	±60	ijk	0.44	±0.02	ab
5A No lime	1.31	±0.23	fgij	2914	±759	i	426	±99	k	0.55	±0.07	bcdefghij
5B Low lime	0.72	±0.23	defgh	3142	±447	i	474	±75	k	0.48	±0.02	abcdefg
6A No lime	1.40	±0.32	fghij	2612	±738	hi	378	±95	jk	0.54	±0.06	bdefgh
6B High lime	0.46	±0.08	de	2337	±176	hi	332	±31	jk	0.45	±0.02	abc
Russell lupin												
1A Low lime	0.99	±0.53	defghi	1042	±333	fg	191	±58	fghi	0.68	±0.09	klmno
1B Low lime	0.05	±0.04	ab	417	±120	abc	61	±20	abc	0.46	±0.02	abcd
2A High lime	0.35	±0.05	d	450	±114	abcde	90	±23	acdef	0.63	±0.01	hjklmno
2B High lime	0.02	±0.006	a	302	±48	ab	50	±8	ab	0.54	±0.04	bcdefghi
3A Low lime	1.42	±0.76	defghij	1173	±558	cefg	210	±94	efghi	0.70	±0.08	no
3B No lime	0.15	±0.09	bc	663	±322	abcd	112	±57	abcd	0.51	±0.03	abcdefg
4A High lime	0.83	±0.39	defg	671	±226	bcdef	138	±46	defgh	0.66	±0.02	kmno
4B No lime	0.11	±0.08	abc	408	±164	a	74	±32	a	0.56	±0.03	defghijkl
5A No lime	0.74	±0.22	defghi	929	±156	fg	177	±25	ghij	0.62	±0.02	hijklmn
5B Low lime	0.05	±0.03	ab	432	±82	abcde	76	±18	abcde	0.54	±0.02	cdefghijk
6A No lime	1.76	±0.68	efghij	1657	±683	gh	287	±89	hijk	0.80	±0.11	o
6B High lime	0.17	±0.08	c	696	±166	cdef	125	±29	defg	0.57	±0.02	ghijklmn
Species	*			*			*			*		
Treatment	*			*			*			*		
Species*Treatment	*			*			*			ns		

ns not significant at *p* < 0.05; * significant at *p* < 0.05.

**Table 3:** Initial soil fertility status of Avenel Station soil (0–0.15 m).

Soil Analysis	Value	By Method of
pH_H2O_	4.7	[43]
Olsen P (μg mL^−1^)	13	[44]
Sulphate Sulphur (μg g^−1^)	17	[45]
Potassium (me 100 g^−1^)	0.59	[46]
Calcium (me 100 g^−1^)	2.1	
Magnesium (me 100 g^−1^)	0.94	
Sodium (me 100 g^−1^)	0.10	
CEC (me 100 g^−1^)	19	[47]
Total Base Saturation (%)	19.6	
Exchangeable Al_CaCl2_ (mg kg^−1^)	25	[48]
Organic matter (%w w^−1^)	11.5	[43]

## Data Availability

The original contributions presented in the study are included in the article, further inquiries can be directed to the corresponding author.
